# Author Correction: Record thermopower found in an IrMn-based spintronic stack

**DOI:** 10.1038/s41467-020-16445-9

**Published:** 2020-05-14

**Authors:** Sa Tu, Timothy Ziman, Guoqiang Yu, Caihua Wan, Junfeng Hu, Hao Wu, Hanchen Wang, Mengchao Liu, Chuanpu Liu, Chenyang Guo, Jianyu Zhang, Marco A. Cabero Z., Youguang Zhang, Peng Gao, Song Liu, Dapeng Yu, Xiufeng Han, Ingrid Hallsteinsen, Dustin A. Gilbert, Mamoru Matsuo, Yuichi Ohnuma, Peter Wölfle, Kang L. Wang, Jean-Philippe Ansermet, Sadamichi Maekawa, Haiming Yu

**Affiliations:** 1 Fert Beijing Institute, BDBC, School of Microelectronics, Beihang University, 100191 Beijing, China; 2 Institut Laue-Langevin, 38042 Grenoble, France; 3 Université de Grenobles-Alpes, and CNRS, LPMMC, 38042 Grenoble, France; 4 Kavli Institute for Theoretical Sciences, University of Chinese Academy of Sciences, 100190 Beijing, China; 5 Department of Electrical Engineering, University of California, Los Angeles, CA 90095 USA; 6 Beijing National Laboratory for Condensed Matter Physics, Institute of Physics, University of Chinese Academy of Sciences, Chinese Academy of Sciences, 100190 Beijing, China; 7 Institute of Physics, Ecole Polytechnique Fédérale de Lausanne (EPFL), 1015 Lausanne, Switzerland; 8 Electron Microscopy Laboratory, School of Physics, Peking University, 100871 Beijing, China; 9 Shenzhen Institute for Quantum Science and Engineering (SIQSE), and Department of Physics, Southern University of Science and Technology (SUSTech), 518055 Shenzhen, China; 10 International Center for Quantum Materials, School of Physics, Peking University, 100871 Beijing, China; 11 Collaborative Innovation Center of Quantum Matter, 100871 Beijing, China; 12 Department of Electronic Systems, Norwegian University of Science and Technology, Trondheim, 7491 Norway; 13 Advanced Light Source, Lawrence Berkeley National Laboratory, Berkeley, CA 94720 USA; 14 Material Science and Engineering Department, University of Tennessee, Knoxville, TN 37996 USA; 15 RIKEN Center for Emergent Matter Science (CEMS), Wako, 351-0198 Japan; 16 Institute for Theory of Condensed Matter, Karlsruhe Institute of Technology, 76049 Karlsruhe, Germany

**Keywords:** Thermoelectrics, Magnetic devices, Spintronics

Correction to: *Nature Communications* 10.1038/s41467-020-15797-6, published online 24 April 2020.

The original version of this Article contained errors in the author list and affiliations.

Author Mamoru Matsuo affiliated with Kavli Institute for Theoretical Sciences, University of Chinese Academy of Sciences, Beijing 100190, China and RIKEN Center for Emergent Matter Science (CEMS), Wako 351-0198, Japan was inadvertently omitted.

Author Yuichi Ohnuma affiliated with Kavli Institute for Theoretical Sciences, University of Chinese Academy of Sciences, Beijing 100190, China was inadvertently omitted.

These have now been corrected in both the PDF and HTML versions of the Article.

