# Supplementary material for: Record thermopower found in an IrMn-based spintronic stack
Source: Nat Commun. 2020 Apr 24;11:2023. doi: 10.1038/s41467-020-15797-6 (PMC7181642; doi:10.1038/s41467-020-15797-6)
Supplement: Supplementary file 1 — Supplementary Information [file 41467_2020_15797_MOESM1_ESM.pdf]

Supplementary Information for

Record thermopower found in an IrMn-based spintronic stack

Tu *et al.*

Supplementary Note 1. Energy dispersive X-ray spectroscopy image

Supplementary Figure 1 shows the energy dispersive X-ray (EDX) spectroscopy images of Ta/IrMn/CoFeB/MgO/Ta structure. The titanium, magnesium, iron and manganese are selectively detected and highlighted by different colours. A Ta/MgO/CoFeB/IrMn/Ta multilayer structure is clearly observed. Slight diffusion of CoFeB into MgO and IrMn might exist, which can be indicated by the colour-coded image.

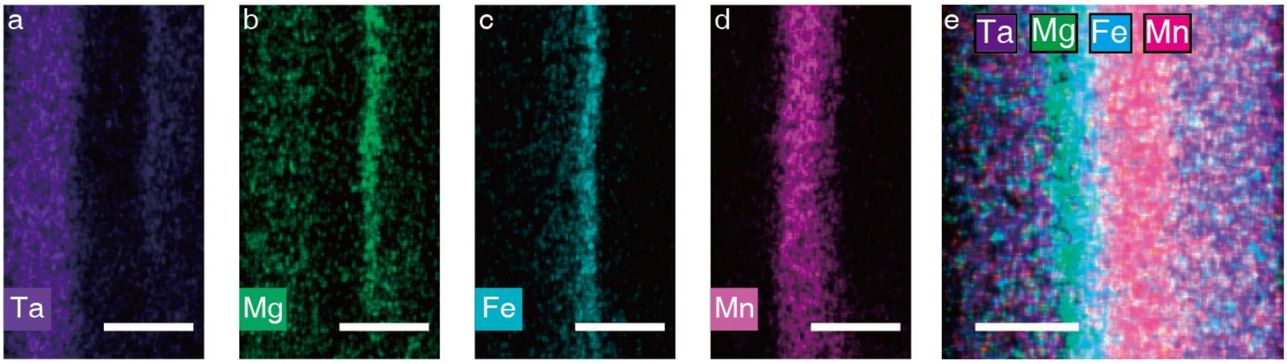

Supplementary Figure 1. Energy dispersive X-ray spectroscopy image corresponding to Fig. 1b in the main text. a-d, High angle annular dark field scanning transmission electron microscopy pictures. Titanium, Magnesium, Iron, and Manganese are highlighted by different colours. **e,** High angle annular dark field scanning transmission electron microscopy picture of the Ta/MgO/CoFeB/IrMn/Ta film cross section. For clarity, we detail the representation of the colours. Purple: Titanium; Green: Magnesium; Blue: Iron; Pink: Manganese. The scale bars of **a-e** are 5 nm.

Supplementary Note 2. Magnetic characterization and Resistivity of the film

The IrMn(t)/CoFeB(0.9)/MgO(2.0) films were grown by magnetron sputtering at room temperature with perpendicular magnetic anisotropy (PMA). The magnetization moment in IrMn(2.8)/CoFeB(0.9)/MgO(2.0) was measured by vibrating sample magnetometer (VSM) at 295 K with an out-of-plane magnetic field. We note a small exchange bias field close to 0.1 mT due to the antiferromagnetic (AFM)-ferromagnetic (FM) interaction between IrMn and CoFeB as shown in Supplementary Figure 2a. Supplementary Figure 2b show the resistivity measurement on IrMn(3.1)/CoFeB(0.9)/MgO(2.0).

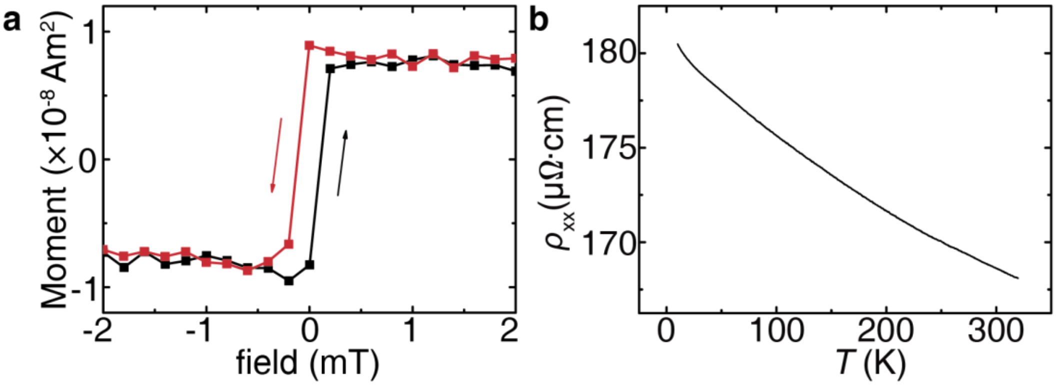

Supplementary Figure 2. Magnetic characterization and resistivity measurement on the film. a, Vibrating sample magnetometer measurement on the sample IrMn(2.8)/CoFeB(0.9)/MgO(2.0) with an out-of-plane magnetic field. **b**, Resistivity measurement on the sample IrMn(3.1)/CoFeB(0.9)/MgO(2.0) from 10 K to 320 K.

Supplementary Note 3. Seebeck coefficient of different samples at room temperature

Based on the single rectangular bar device in Fig. 1a, we conducted the Seebeck coefficient measurements in IrMn(*t*)/CoFeB(0.9)/MgO(2.0) samples at room temperature. Supplementary Figure 3a-l present the results of ΔT dependence of Seebeck voltage in all samples, namely, with 0.6 nm, 1.0 nm, 1.7 nm, 2.0 nm, 2.5 nm, 2.8 nm, 3.1 nm, 3.2 nm, 3.4 nm, 4.0 nm thick IrMn(*t*)/CoFeB(0.9)/MgO(2.0), CoFeB(0.9)/ MgO(2.0) and IrMn(3.1)/MgO(2.0). A polarity change the Seebeck coefficient was observed in samples of 0.6 nm in thickness, which includes only two atomic planes of IrMn. The sample with 1 nm of IrMn also has a negative Seebeck coefficient, but it is nearly zero. All the figures present a linear dependence between Seebeck voltage and ΔT. The largest Seebeck coefficient value 390($\pm$10) μV K^{-1} has been observed in the sample with 3.1 nm thick IrMn at room temperature (Supplementary Figure 3g).

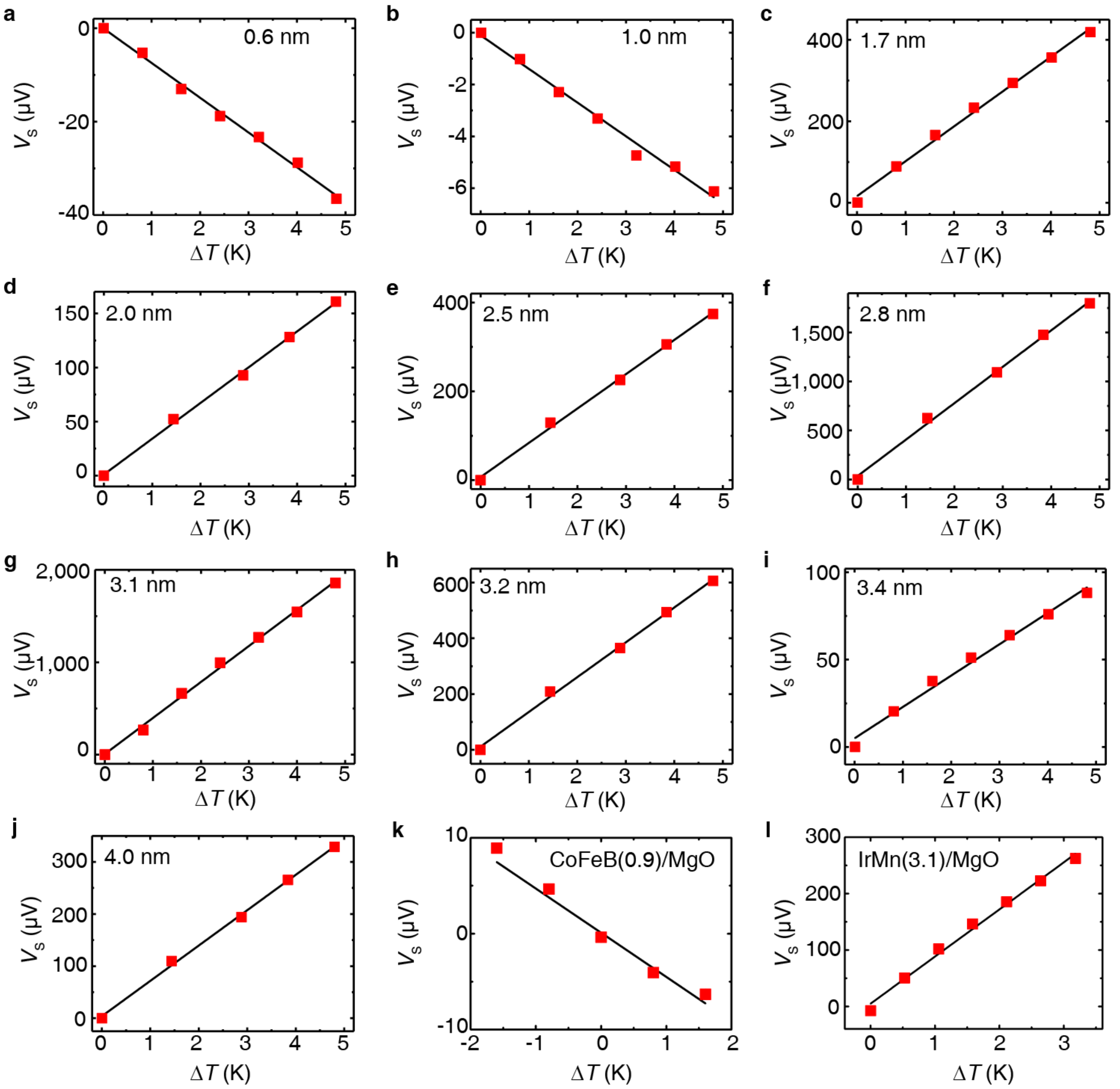

Supplementary Figure 3. Seebeck coefficients of single rectangular bar structure with different IrMn thicknesses at room temperature. a-l show the thermopower as a function of ΔT, from which one could calculate the Seebeck coefficient, on different samples. (**g** has been shown in inset of Fig. 1c)

Supplementary Note 4. Estimation of the figure of merit

To judge the real potential of the thin film, we have estimated the figure of merit. By taking the averaged electrical resistivity ρ of the whole CoFeB/IrMn thin film as 220 μΩ cm from Ref. 41 in the main text and the thermal conductivity $\kappa = 9\ \mathrm{W\ m^{-1}\ K^{-1}}$ from Ref. 33 in the main text, we estimated the figure of merit [$zT = S^2T/\rho\kappa$] for the sample IrMn (3.1 nm)/CoFeB (0.9 nm)/MgO (2.0 nm) [S(RT)= 390 $\mu\mathrm{V\ K^{-1}}$] at the room temperature (295 K) to be 2.2. This is quite a large value but this is a rough estimate and it does not take into account the heat flow in the substrate.

Supplementary Note 5. Single rectangular bar sample fabrication and temperature gradient measurements

In order to investigate the thickness dependence of the Seebeck coefficient in IrMn thin films at room temperature, the films were patterned into single rectangular bar devices using the techniques of lithography and ion beam etching. Fig. 1a shows an image of the device structure. The two Peltier elements along the x-axis were put on the rear side of the sample to induce an in-plane temperature gradient and enable Seebeck voltage measurements. An infrared thermal camera was used to observe the temperature difference on the sample surface. As shown in Supplementary Figure 4, we can get a temperature difference about 4.8 K in the length of 8 mm along the sample surface. The colour bar was the temperature distribution taken by the infrared thermal camera.

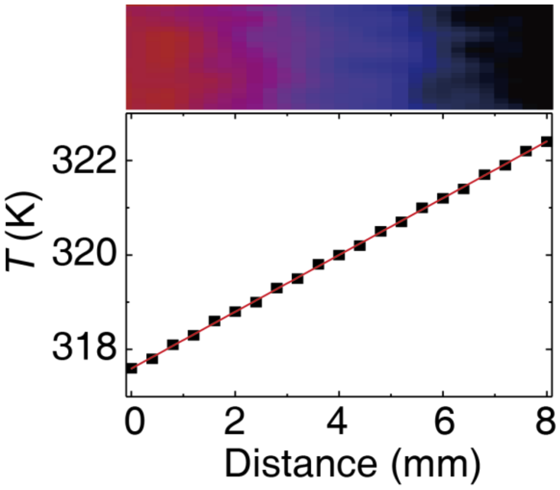

Supplementary Figure 4. The details of the temperature gradient. Temperature calibration by using an infrared thermal camera. Top: infrared thermal cameral image of the sample surface. Bottom: temperature distribution along the sample surface.

Supplementary Note 6. Layout of the meandering thermoelectric device

In order to investigate the temperature dependence of the Seebeck coefficient, the films were patterned into meandering devices using lithography and ion beam etching technique. The micrograph of the meandering device is shown in Supplementary Figure 5. One side of the device can be heated up by applying a current due to Joule heating in the gold heater. The size of each gold stripe is 4 μm in width, 2,000 μm in length and 120 nm in thickness. Each stripe A consists of an IrMn/CoFeB/MgO stack. The size of this part is 12 μm in width, 1,500 μm in length and nearly 10 nm in thickness. Each stripe B is made of gold, with a width of 10 μm, a length of 1,500 μm and a thickness of 120 nm. The distance between part A and part C (the Joule heater) is 80 μm and the gap between part A and part B is 10 μm. Part A and B connect from end to end, so that the Seebeck voltages add up. The

thermoelectric voltage in stripe B is tiny. As a result, compared to typical thermoelectric signals, a giant voltage signal was obtained. To measure Seebeck voltage, the lock-in amplifier was connected to electrodes 3 and 5. In this case, 14 effective stripes contributed to the measured Seebeck voltage.

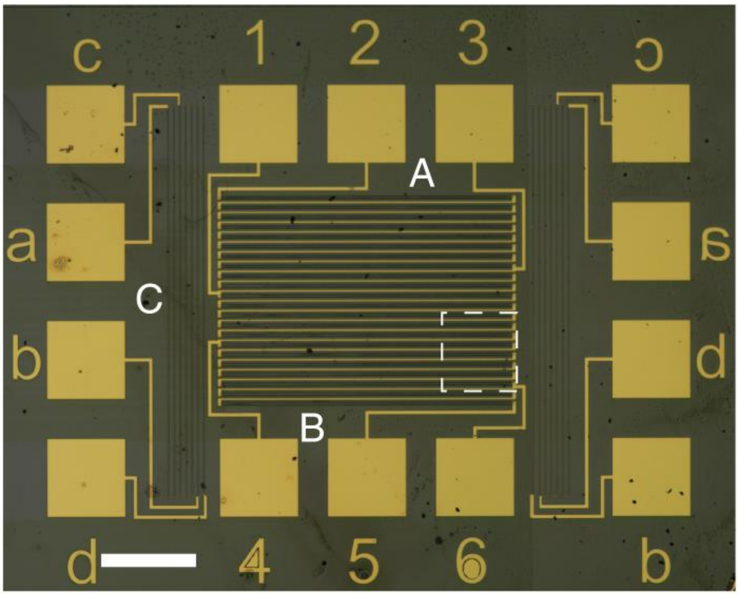

Supplementary Figure 5. The details of the sample fabrication. Optical micrograph of the meandering device structure for the Seebeck experiment. Part A: sample stripes consist of Ta/MgO/CoFeB/IrMn/Ta. Part B: gold stripes as connections for samples stripes. Part C: gold stripes as a Joule heater. Actual thermoelectric device with meandering structures are circled, corresponding to Fig. 2a. The scale bar is 200 μm.

Supplementary Note 7. Temperature-dependent measurements of the thermopower

Based on the large thermopower obtained for the meandering device at room temperature, we also measured the temperature dependence of thermopower in IrMn(*t*)/CoFeB(0.9)/MgO(2.0) samples. Supplementary Figure 6a-i are the results of temperature dependence of thermopower in rest of all samples, namely, with 0.6 nm, 1.0 nm, 1.7 nm, 2.0 nm, 2.5 nm, 3.1 nm, 3.2 nm, 3.4 nm, and 4.0 nm thick IrMn. Strong temperature-dependent thermopower in different samples is observed. A temperature peak is found at the thicknesses of IrMn above 1.0 nm, when the temperature window is sufficiently large.

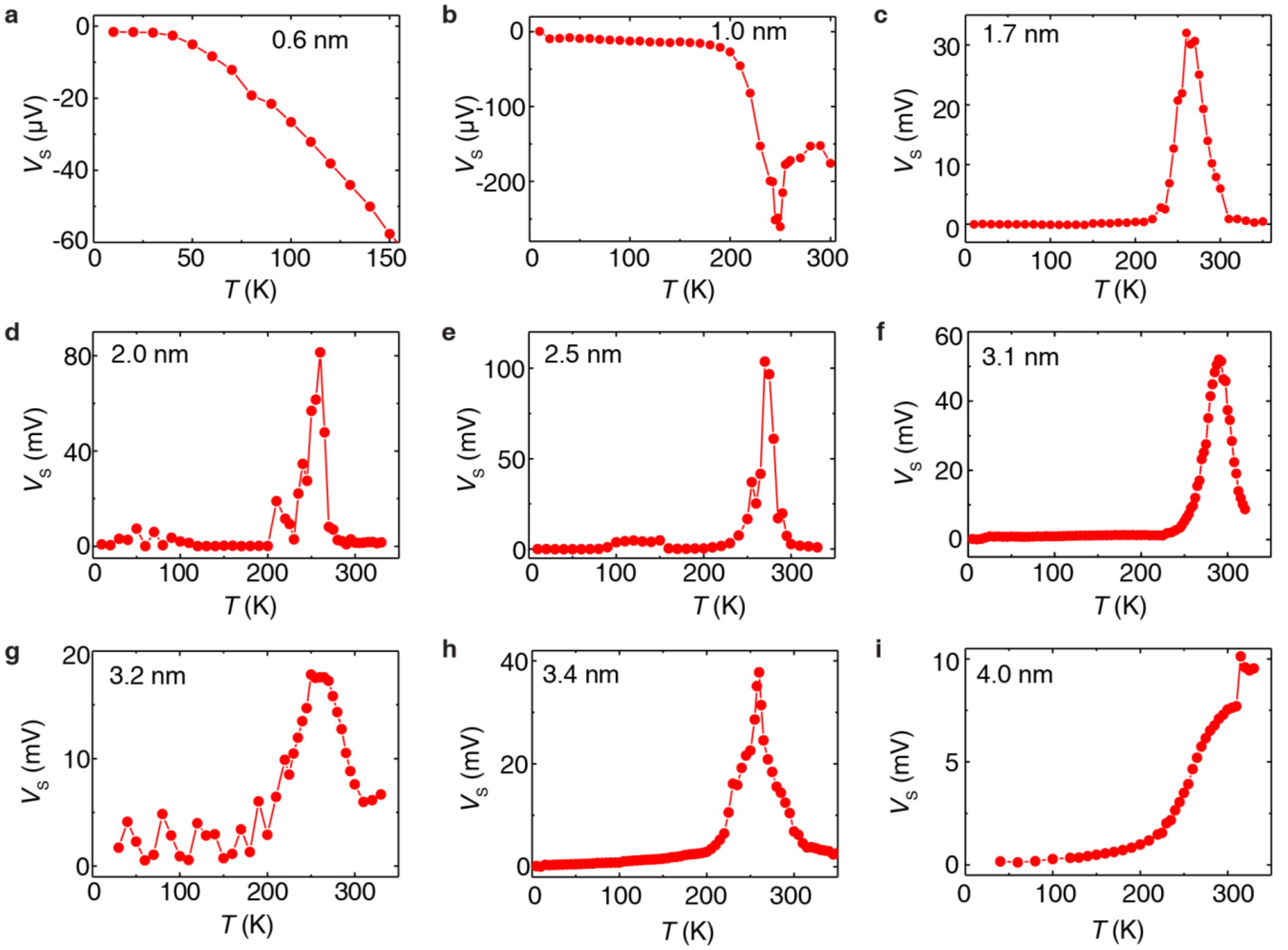

Supplementary Figure 6. Thermopower depends on different environment temperatures. a-i show temperature dependence of thermopower from 10 K to 320 K on different samples corresponding to 0.6 nm, 1.0 nm, 1.7 nm, 2.0 nm, 2.5 nm, 3.1 nm, 3.2 nm, 3.4 nm, and 4.0 nm thick IrMn in IrMn(t)/CoFeB(0.9)/MgO(2.0).

Supplementary Note 8. Giant thermopower in a nano-scale meandering device

In order to generate large thermopower in our meandering device, we fabricated a nano-scale meandering device on IrMn (2.8)/CoFeB/MgO thin film utilizing a higher density of thermopiles and larger heating power. The size of each IrMn trilayer stripe is 800 nm in width, 400 μm in length and nearly 10 nm in thickness. The period of each IrMn trilayer stripe is 3.2 μm. The nano-scale meandering device connected 20 IrMn stripes in total for the thermopower accumulation. The Scanning Electron Microscope (SEM) image of the meandering device is shown in Fig. 2b. An AC current was applied at a frequency of 17 Hz and the thermopower was measured with a lock-in amplifier at twice of the frequency. The thermopower as a function of the current is shown in Supplementary Figure 7.

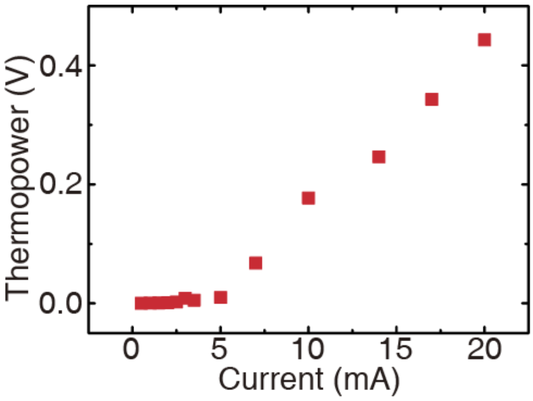

Supplementary Figure 7. Thermopower measurement. Thermopower as a function of the current on the nano-scale meandering device at 330 K. A heating current of up to 20 mA is applied.

Supplementary Note 9. Thickness-dependent Seebeck coefficient at critical temperature

On these magnetic multilayers with perpendicular magnetized anisotropy, we concluded the thickness dependence of Seebeck coefficient at the critical temperature (T_{crit}). The results are shown in Supplementary Figure 8. The largest Seebeck coefficient $S(T_{\mathrm{crit}})$ is more than 1.0 mV K^{-1} in sample with t_{AFM} = 2.5 nm.

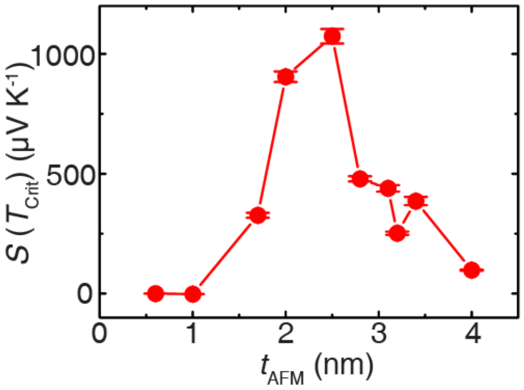

Supplementary Figure 8. Thickness dependence of the Seebeck coefficient at critical temperature. The Seebeck coefficient at the critical temperature as a function of IrMn thickness t_{AFM}. The error bar is extracted from the results difference of measurements which were repeated ten times.

Supplementary Note 10. Temperature-dependent susceptibility measurements

In this section, we report the DC susceptibility measured at different temperatures on the pure IrMn samples. For comparison, four different thicknesses of samples were characterized, namely, 2.0 nm, 2.5 nm, 3.0 nm and 4.0 nm, as shown in Supplementary Figure 9. The samples were measured in a standard Quantum Design Magnetic Property Measurement System (MPMS), allowing measurements of magnetization **M** (**H**, *T*) (temperature scans, hysteresis etc.) for temperatures from 2 K to 400 K and in a range of magnetic fields from -5 T to 5 T. The measurement procedure is the following: first of all, the prepared samples are mounted in a capsule and inserted into the magnetometer chamber of the MPMS and cooled down to 5 K without magnetic field. Second, the magnetization is measured as a function of temperature raising from 5 K to 380 K with a fixed 1 T out-of-plane magnetic field. As a result, we verified that the inflection points of the susceptibility depending on temperature on the pure IrMn samples show the same trend as that of IrMn/CoFeB/MgO thin films. The critical temperatures extracted from Supplementary Figure 9a-d are about 270 K, 280 K, 290 K and 310 K on 2.0 nm, 2.5 nm, 3.0 nm and 4.0 nm pure IrMn thin film, respectively.

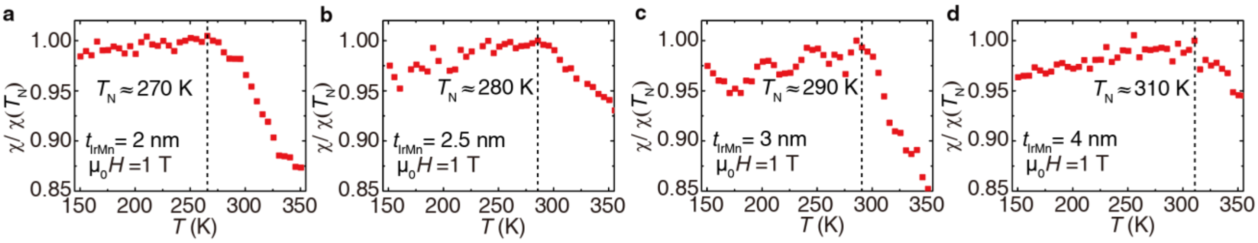

Supplementary Figure 9. Temperature-dependent susceptibility measurements. a-d, 2.0 nm, 2.5 nm, 3.0 nm and 4.0 nm pure IrMn samples, sweeping the temperature from 150 K to 350 K, respectively. In all measurements an out-of-plane magnetic field of 1 T was applied.

Supplementary Note 11. Fitting parameters for thickness dependence of the critical temperature

There is an alternative method to probe the magnetic phase transitions on antiferromagnets, according to Ref. 24 in the main text. Spin pumping efficiency in NiFe/IrMn thin films is enhanced around the magnetic phase transition. The critical temperature ($T_{\mathrm{crit}}^{\mathrm{IrMn}}$) is found to have a strong thickness dependence of the IrMn layer. For $t_{\mathrm{IrMn}} < n_0$,

$$T_{\mathrm{crit}}^{\mathrm{IrMn}}(t_{\mathrm{IrMn}}) = T_{\mathrm{N}}^{\mathrm{IrMn}}(\mathrm{bulk})\frac{t_{\mathrm{IrMn}}-d}{2n_0} \quad . \tag{1}$$

For $t_{\mathrm{IrMn}} > n_0$,

$$T_{\mathrm{crit}}^{\mathrm{IrMn}}(t_{\mathrm{IrMn}}) = T_{\mathrm{N}}^{\mathrm{IrMn}}(\mathrm{bulk})[1-\frac{n_0+d}{2t_{\mathrm{IrMn}}}] \quad . \tag{2}$$

where $T_{\mathrm{N}}^{\mathrm{IrMn}}$ (bulk) is the Néel temperature of the IrMn bulk films, n_0 is a spin-spin correlation length, and *d* is the interatomic distance. The parameters utilized in Fig. 3a in main text are $T_{\mathrm{N}}^{\mathrm{IrMn}}$=400 K, n_0=1.2 nm and *d*=0.1 nm. The discrepancy may be induced by different IrMn targets and crystal orientation of the thin films. The fitting curve is shown in Fig. 3a of the main text.

Supplementary Note 12. X-ray magnetic linear dichroism in IrMn/CoFeB/MgO multilayers

In this section, we report the X-ray magnetic linear dichroism (XMLD) and X-ray absorption spectroscopy (XAS) measurements in the IrMn(3.2)/CoFeB(0.9)/MgO(2.0) and IrMn(4.0)/CoFeB(0.9)/MgO(2.0) thin films at 200 K and 300 K. The XAS/XMLD measurements are performed in a normal geometry with the linear polarization oriented along orthogonal Cartesian axes in the plane of the films. This orientation captures differences in the in-plane orbital occupancy. Since the sample is field cooled, AFM ordering is expected to be uniform over large areas, resulting in an asymmetric occupancy of the orbitals and a net XMLD signal. At the blocking temperature, local ordering persists, but long-range ordering is lost due to thermally activated domain reorientation. The X-ray signal is captured over a sample area ≈ 75 $\mu\mathrm{m}^2$, and reports the average orbital occupancy over that area. Above the blocking temperature, since each domain is expected to be randomly oriented in-plane, the difference of signal will be reduced to zero.

To reduce the effects of drift and other chronic noise sources, we performed twenty sequential measurements with alternating linear polarization (A/B/A/B/etc.) at each temperature. Each of these scans were corrected for vertical offsets and linear drift in intensity, normalized, then measurements with the same polarization were averaged. Supplementary Figure 10 shows the XAS signal (the sum of both polarizations) and the XMLD signal (the difference of the polarizations) scaled by $10\times$. Data is presented for the Mn $L_{2,3}$ edge IrMn(3.2)/CoFeB(0.9)/MgO(2.0) and IrMn(4.0)/CoFeB(0.9)/MgO(2.0) thin films at 200 K and 300 K. The XAS results show the expected spectra for metallic Mn and change very little between sample or temperature. The XMLD signal shows a characteristic negative/positive feature pair at 200 K, occurring at ≈ 640 eV, as reported previously (Ref. 46, 47 in the main text). indicating AFM ordering in IrMn. At 300 K, the 3.2 nm film has no distinguishable feature pair, while the 4.0 nm has a much weaker feature. The signal presented in the main text Fig. 3b is derived by twice-integrating the XMLD feature between 636 eV and 642 eV, capturing the intensity of the feature pairs.

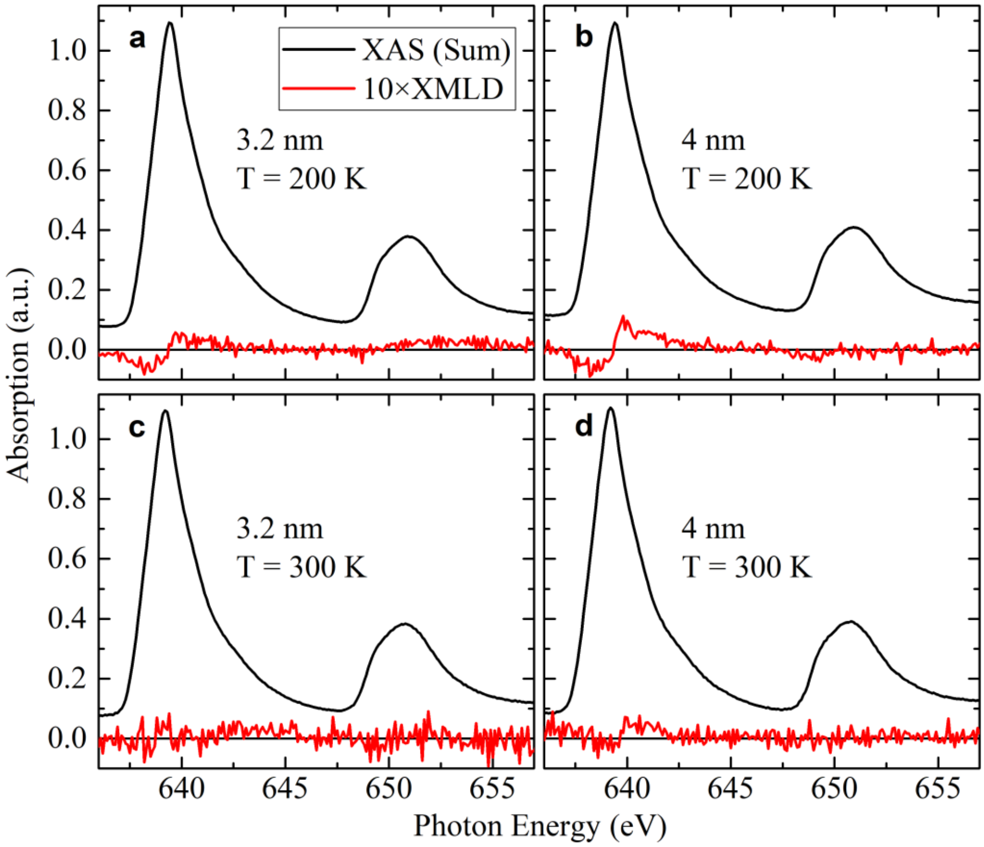

Supplementary Figure 10. X-ray magnetic linear dichroism (XMLD) and X-ray absorption spectroscopy (XAS) measurements. The XMLD and XAS signals for the IrMn(3.2)/CoFeB(0.9)/MgO(2.0) and IrMn(4.0)/CoFeB(0.9)/MgO(2.0) thin films at (**a**, **b**) 200 K and (**c**, **d**) 300 K, respectively.

Supplementary Note 13. Theory and discussion for thermopower near the critical temperature

To understand the results from a theoretical point of view, there are two key questions: first, why are the critical temperatures so much reduced from the bulk (as shown in Fig. 3b) in thin samples and second, what is the origin of the sharp increase in thermopower around the critical temperature? In Ref. 23, Frangou *et al.* modelled the decrease in critical temperatures for thin films by the loss of transverse couplings as the geometry changes from three to two dimensions. In slightly more general terms, strong reduction of the ordering temperature is expected due to a dimensional crossover from a nearly isotropic Heisenberg antiferromagnet to the quasi two-dimensional antiferromagnet, where anisotropies must limit the effects of an increased density of long wavelength spin waves. Past calculations of electronic transport in the presence of magnetic fluctuations (Ref. 55 in the main text), including the effects of critical slowing down of fluctuations near the transition ultimately predicted a rather weak singularity proportional to the internal energy (Ref. 56 in the main text) in the electrical resistivity (Supplementary Figure 2b). For the thermopower, both charge and heat currents must be considered. The previous two works (Ref. 57, 58 in the main text) argued for the importance of both inelastic as well as elastic scattering, where perturbation in the coupling between the conduction electrons and the magnetic moments is considered. The scattering time is calculated from spin fluctuations that contribute to the thermopower and whose inverse is proportional to the dynamical structure factor for momentum transfer across the Fermi surface. If this is a small correction to the total scattering rate, it contributes a term linear in the enhanced magnetic susceptibility to the thermopower close to the critical temperature, but reduces the absolute value. As discussed in the main text, Peter Wölfle (Ref. 53), has recently proposed a theory based on proximity to a quantum critical

point, which predicts a strong enhancement of the Seebeck coefficient, particularly in two dimensions. This theory requires inclusion of impurity scattering, which itself can be enhanced in the film geometry, and predicts a peaked form, with power law increase cut off close to the ordering temperature that resembles our measurements. Of course, we cannot rule out the possibility that the variation comes from the other terms in Equation (2): the density of states or the Fermi velocity. While it is not clear which effect dominates, or if all contribute, even in a well-studied metal such as chromium (Ref. 49, 50 in the main text), we can argue that the enhanced spin fluctuations are very important in our films.

Supplementary Note 14. The influence of the CoFeB layer on the Seebeck coefficient

To investigate the influence of the CoFeB layer on the Seebeck coefficient, we performed a temperature-dependent comparison experiment of the thermopower to observe the difference of thermopower between the multilayer samples with and without the CoFeB layer when the thickness of IrMn is 3.1 nm. As shown in Supplementary Figure 11, one could find that the peak points of the temperature-dependent measurements of the thermopower are both around approximately the same temperature (≈290 K), indicating that the CoFeB layer does not influence much the phase transition of IrMn. In addition, it is worth noting that the thermopower on the sample with the CoFeB layer is almost seven times larger than the sample without the CoFeB layer at the peak point.

As we have mentioned in the main text, there could be some possible reasons to explain this observation. Firstly, the contribution of magnon/spin current should be considered. The magnon/spin current at the interface of can contribute to the Seebeck effect as studied previously (Ref. 28,29,31,33,66,67 in the main text). In our case, the magnon/spin current will contribute into the spin Seebeck effect induced by out of plane temperature gradient which can't be disregarded in the measurements. The spin Seebeck effect can also be enhanced around its critical temperature, but the amplitude is typically in the range of the hundreds of nV which is not comparable to the Seebeck effect observed in this work up to mV. Therefore, the magnon/spin transport mediated spin Seebeck effect can contribute to the large Seebeck coefficient but is not the key role. Moreover, the interface of IrMn/CoFeB could be important since the CoFeB is a ferromagnet. Due to the magnetic proximity effect, the spin moment in the AFM might be influenced and generate effective internal magnetic field, which can further affect the spin fluctuations in the AFM layer itself. As we have discussed in the main text, the Seebeck effect could be directly related to the spin fluctuations. Therefore, the magnetic proximity-induced spin fluctuations in AFM/FM interface can contribute to the large Seebeck effect.

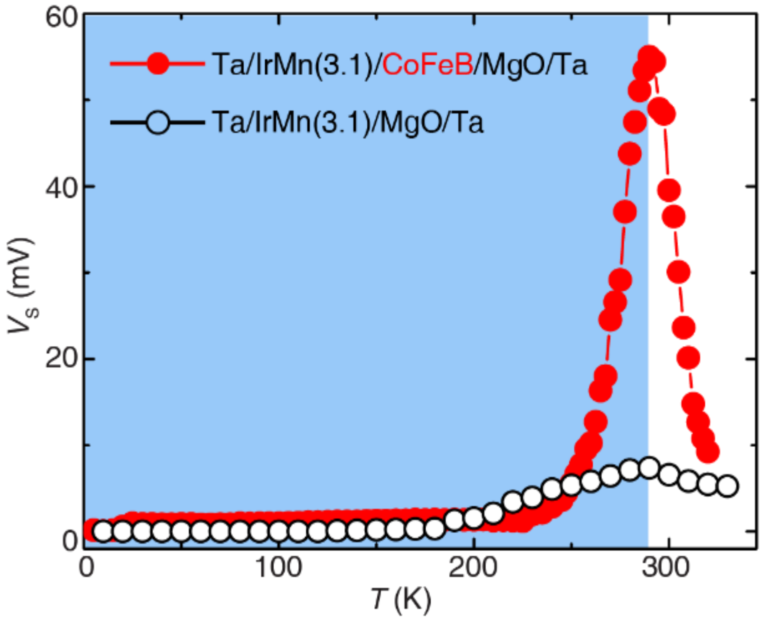

Supplementary Figure 11. Direct comparison of the Seebeck voltage measured as a function of temperature on multilayer samples with (red dots) and without (black circles) CoFeB layer. The IrMn thickness is fixed at 3.1 nm for both samples. Temperature dependence of thermopower for both samples show maxima around 290 K, close to room temperature. The peak amplitude with CoFeB is about 7 times larger than without CoFeB.

Supplementary Note 15. Anomalous Nernst coefficient of different samples at room temperature

In addition to studying the Seebeck effect, we also performed the anomalous Nernst coefficient measurements in IrMn(t)/CoFeB(0.9)/MgO(2.0) samples at room temperature. Supplementary Figure 12a-k present the field dependence of anomalous Nernst voltage in all samples, namely, with IrMn thickness of 0.6 nm, 1.0 nm, 1.7 nm, 2.0 nm, 2.5 nm, 2.8 nm, 3.1 nm, 3.2 nm, 3.4 nm, 4.0 nm. The largest anomalous Nernst coefficient of 2.3 ($\pm$0.2) $\mu V\ K^{-1}$ is observed in the sample with 3.1 nm-thick IrMn at room temperature.

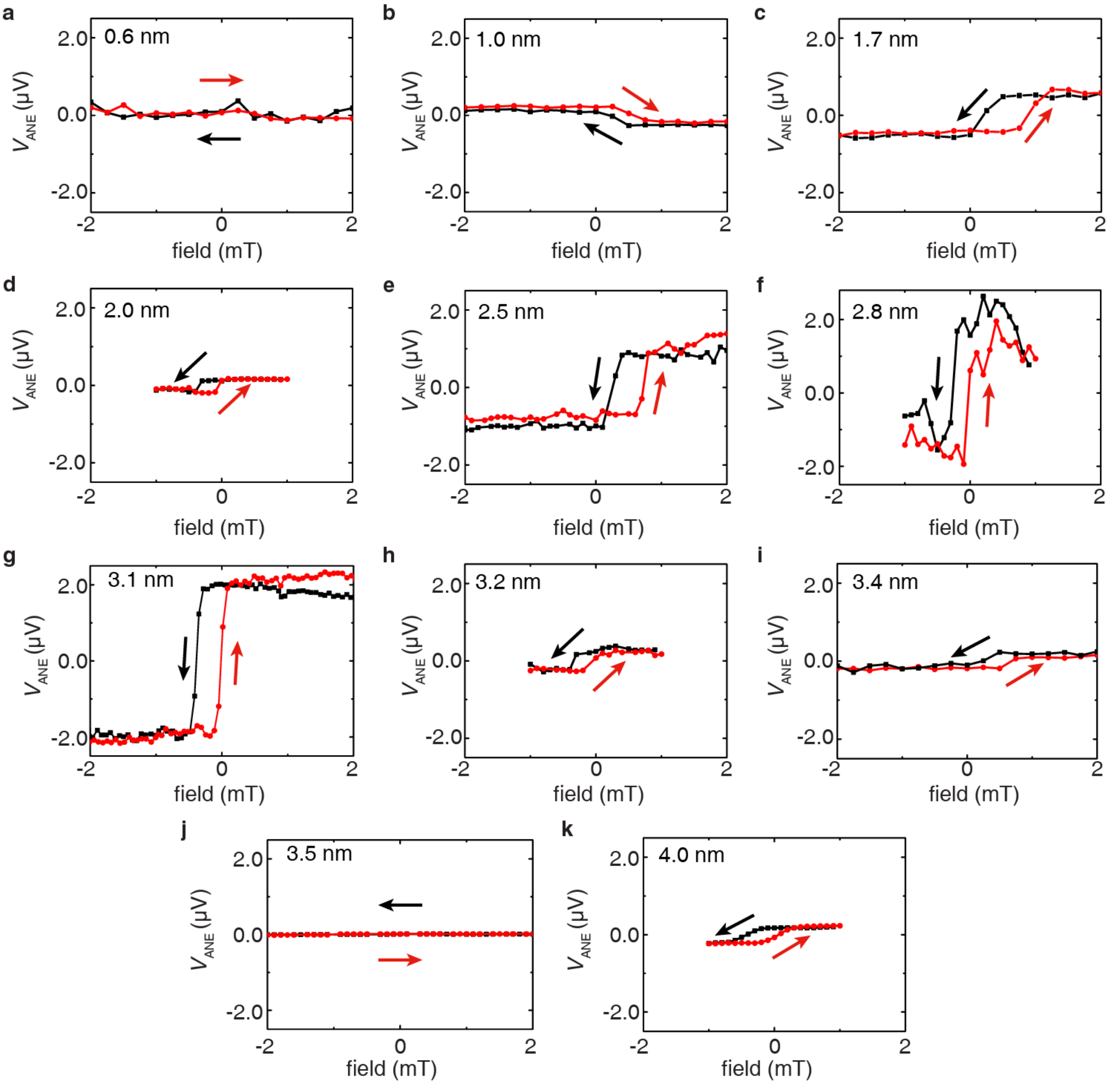

Supplementary Figure 12. Anomalous Nernst voltages of single rectangular bar structure with different IrMn thicknesses at room temperature. a-k show the anomalous Nernst voltages as a function of magnetic field, from which one can calculate the anomalous Nernst coefficient on different samples.

Supplementary Note 16. Magnetothermopower on IrMn(3.1 nm)/CoFeB(0.9 nm)/MgO(2.0 nm)

The magnetothermopower (MTEP) was measured on the structure IrMn(3.1 nm)/CoFeB(0.9 nm)/MgO(2.0 nm), which has the largest Seebeck coefficient at room temperature. The MTEP shown in Supplementary Figure 13 was measured with an external field sweeping along the *z* axis (normal to the sample plane). The MTEP shows a similar hysteresis loop as the ANE in the sense that the switching fields of the ferromagnetic CoFeB layer agree well with magnetization measurements (see Supplementary Figure 2a). Both the ANE and MTEP measurements reveal that the CoFeB layer also plays a role in the thermoelectric properties of the multilayer structures, in addition to the critical influence from the phase transition of AFM IrMn layer.

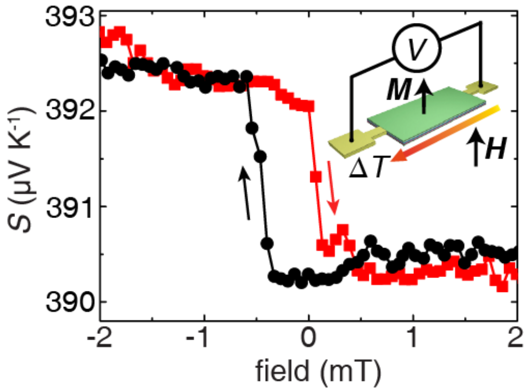

Supplementary Figure 13. Magnetothermopower (MTEP) of IrMn(3.1 nm)/CoFeB(0.9 nm)/MgO(2.0 nm) while sweeping an out-of-plane field. Magnetothermopower on the single rectangular bar sample IrMn(3.1)/CoFeB(0.9)/MgO(2.0) with an out-of-plane magnetic field and an in-plane ΔT equals 4.8 K at room temperature. Inset is an illustrative diagram of MTEP.
